# Clinical and Microbiological Determinants of Outcome in *Staphylococcus aureus* Bacteraemia

**DOI:** 10.1155/2010/654858

**Published:** 2010-03-16

**Authors:** James Price, Gillian Baker, Ian Heath, Karen Walker-Bone, Marc Cubbon, Sally Curtis, Mark C. Enright, Jodi Lindsay, John Paul, Martin Llewelyn

**Affiliations:** ^1^Medical Research Building, Brighton and Sussex Medical School, University of Sussex, Falmer BN1 9PS, UK; ^2^Department of Microbiology and Infection, Brighton and Sussex Hospitals NHS Trust, Eastern Road, Brighton BN2 5BE, UK; ^3^Faculty of Medicine, St Mary's Campus, Imperial College, London W2 1PG, UK; ^4^St Georges Hospital Medical School, Cranmer Terrace, London SW17 0RE, UK

## Abstract

*Staphylococcus aureus* bacteraemia (SAB) is commonly complicated by metastatic infection or relapse after treatment. *Objectives*. The study aim was to determine the role of bacterial, host, and management factors in development of complicated SAB. *Methods*. A prospectively-conducted observational study gathered data on predisposition, management and outcome of 100 consecutive SAB cases. Antibiotic susceptibilities and genetic lineage of bacterial isolates were determined. Further clinical and microbiological data were gathered on two retrospective series from 1999–2000 (*n* = 57) and 2004 (*n* = 116). *Results*. In the prospective cases, 27% met our definition of complicated disease. Expressed as RR and 95% CI, complicated disease was associated with diabetes (1.58, 1.00–2.48), injecting-drug use (5.48, 0.88–33.49), community-onset of symptoms (1.4, 1.02–1.92), and symptom duration ≥48 hours prior to starting effective antibiotic therapy (2.10, 1.22–3.61). Uncomplicated disease was associated with the presence of a central line (0.69, 0.55–0.88) and prompt removal of a primary focus (0.71, 0.57–0.90). Neither methicillin resistance nor genetic lineage was associated with complicated disease, but methicillin resistance was associated with higher mortality. *Conclusions*. This study demonstrates that clinical rather than microbial factors are the major determinants of SAB outcome and underscores the importance of early treatment.

## 1. Introduction

*Staphylococcus aureus *bacteraemia (SAB) is one of the commonest forms of healthcare associated infection. Among causes of bacteraemia, *S. aureus* is notable for how commonly it is complicated by the development of secondary foci of infection and relapse. Reported rates of complicated disease range from 12.5% to 53% depending on case definition and the population studied [1–4]. Clinical risk factors for development of complicated bacteraemia are well defined and include community onset of disease and suboptimal medical management [3, 5]. Few data exist on the importance of microbial factors, other than methicillin resistance, in determining the outcome of *S. aureus* infection. Previous studies have compared strain genotype from invasive and noninvasive disease [6, 7] but we are aware of only one previous study assessing the impact of genotype on outcome of bacteraemia [8]. The aim of this study was to determine the relative impact of clinical and microbiological factors on risk of developing complicated SAB.

## 2. Methods

Brighton and Sussex University Hospitals NHS Trust (BSUH) is an 820-bed Teaching Hospital with a high SAB rate. A prospectively conducted observational study of all new cases of SAB was conducted at BSUH between August 2006 and May 2007. The sampling time-frame was determined by the tenure of the trainee microbiologist running the study. Anonymised clinical data were gathered from the case notes on the day of the first clinical review and at a follow-up by the infectious diseases team. 

 A retrospective evaluation was also performed of two cohorts of consecutive SAB cases from which isolates had been archived as part of a previous unpublished study. The first consisted of SAB episodes during 1999-2000. The second consisted of SAB episodes between January and May 2004. Clinical data were collated from case notes and laboratory and administrative databases.

## 3. Ethics Statement

The study was approved by the Brighton and Sussex University Hospital NHS Trust Research and Development office as a service evaluation gathering anonymised, routine clinical data from patient records and not requiring formal ethical review.

## 4. Case Definitions

SAB was defined as at least one positive blood culture for *S. aureus*. A primary focus of infection was defined as the likely source of the bacteraemia according to clinician assessment. Complicated bacteraemia was defined as the development of either a secondary deep focus of infection (on the basis of clinical or microbiological evidence) or relapse of bacteraemia (a second episode of SAB within three months of the first episode). SAB was considered to be community-associated using the CDC definition (diagnosis <48 hours of admission, no previous admission to hospital within twelve months, no previous MRSA positive cultures, and no long-term indwelling devices) [9]. Community-onset disease was defined as SAB diagnosed within 48hrs of admission but not fulfilling criteria for community-associated disease. All SAB diagnosed after 48hrs of admission was defined as hospital onset. Initial antibiotic management was defined as having been “effective” if the patient received parenteral therapy with a beta-lactam antibiotic or a glycopeptide (depending on sensitivities) either alone or in combination with other agents.

## 5. Microbiological Analysis

Blood culture isolates were identified as *S. aureus* on the basis of colony morphology, Gram's staining, and a positive coagulase test. Antibiotic susceptibility was performed by disk diffusion analysis according to British Society for Antimicrobial Chemotherapy guidelines and vancomycin minimum inhibitory concentrations (MIC) were determined by *E*-test. 

 Bacterial isolates from the prospective study were genotyped using a restriction-modification (RM) test. This assigns *S. aureus* isolates to six of the ten major *S. aureus* lineages, clonal complex (CC) 1, 5, 8, 22 (corresponding to MLST 22, predominantly EMRSA-15), 30 (corresponding to MLST 30 and 36, predominantly EMRSA-16), and 45 (corresponding to MLST 45, 4, and 54) [10, 11]. Briefly, RM typing exploits the close relationship between variations in the two *hsdS* genes and *S. aureus* lineage. Combinations of eight primers are used in three PCR reactions to produce products of different sizes [11]. Genes encoding Panton-Valentine Leukocidin (PVL), Toxic Shock Syndrome Toxin (*tst*), and enterotoxins (*sea* and *sej*) were sought by PCR using published primers [12–14].

## 6. Statistical Analysis

Data were analysed using SPSS 15.0.0 and Minitab 15. Associations between complicated disease and the variables described were univariately assessed using the Fisher's exact test for proportions and the Mann-Whitney *U* test for nonparametric continuous variables. Probability values ≤0.05 were considered significant. For categorical variables the relative risk (RR) and 95% CI were calculated. Variables statistically significant in the univariate analysis were analysed in a multivariate logistic regression model to assess which were the significant predictors of complicated disease.

## 7. Results

One hundred SAB cases occurred during the study period. Fifty-two (52%) were MRSA, equating to an overall rate of 6.68 SAB cases and 3.47 MRSA cases per/10 000 bed days. The national average MRSA bacteraemia rate for acute trusts at the start of the study period was 1.77/10 000 bed days

## 8. Patient Characteristics and Clinical Management

Fifty-nine (59%) of SAB cases were male and the median age was 65.5 years. Twenty-seven (27%) fulfilled the criteria for complicated SAB (Table 1); 24 (24%) had secondary site infections, 6 (6%) had relapse and 3 had both. On univariate analysis, complicated bacteraemia was associated with community-onset disease (RR = 1.4, 95% CI: 1.02–1.92, *P* = .018), diabetes mellitus (RR = 1.58, 95% CI: 1.00–2.48, *P* = .021), and injecting drug use (IDU) (RR = 5.48, 95% CI: 0.88–33.49, *P* = .001). The presence of a central line was associated with a reduced risk of complicated disease (RR = 0.69, 95% CI: 0.55–0.88 *P* = .003). 

In 77% of cases a primary source of bacteraemia was apparent. Although the presence or absence of a primary source did not correlate with complicated disease, removal of an identified focus within 72 hours was associated with a lower risk of complicated disease than when the focus was removed beyond this time or not removed at all (RR = 0.71, 95% CI: 0.57–0.90, *P* = .006). Bacteraemic patients who were symptomatic for >48 hours before effective antibiotic therapy was started were at higher risk of complicated disease (RR = 2.10, 95% CI: 1.22–3.61, *P* = .015). There was no relationship between duration of effective intravenous therapy <2 weeks and complicated disease (RR = 1.07, 95% CI: 0.84–1.37, *P* = .641). The risk of death in patients with complicated disease was higher (RR = 1.29, 95% CI: 0.99–1.70, *P* = .057). 

 In the multivariate analysis, both forward and backward likelihood ratio (LR) approaches selected diabetes mellitus, IDU and Time to Removal of Primary Focus in their final model, where backward LR also included time to initiation of effective antibiotics. Diabetes mellitus and IDU were significant at the 5% level for both approaches with *P*-values in the backward LR model of.005 and.004, respectively.

## 9. Retrospective Study

The two retrospective case series comprised 57 bacteraemias in 57 patients in 1999-2000 and 116 bacteraemias in 114 patients in 2004 (Table 2). Good quality basic demographic and microbiological data were available on the great majority of cases but full case notes were available for only 22/57 (38.6%) and 73/116 (62.9%), respectively. The rate of complicated disease among evaluable patients in the 2004 series (20/73 (27.4%)) was comparable with that found in the prospective series and higher than that found in the 1999-2000 series (2/22 (9.1%)) but this did not reach statistical significance (*P* = .074). There was a trend towards more diabetes mellitus and intravenous drug use in the 2004 series compared with the 1999-2000 series, 27.4% versus 9.1% (*P* = .06) 9.6% versus 0% (*P* = .15), respectively.

## 10. Microbial Analysis

Data on antibiotic sensitivity of the *S. aureus *isolates were available on all isolates from the clinical records. The number of bacteraemias caused by MRSA was 30 (52.6%) in 1999, 69 (59.5%) in 2004, and 52 (52%) in 2006. Forty-five of patients received vancomycin as treatment for their SAB. These isolates were all confirmed as vancomycin sensitive with vancomycin MIC by *E*-test ranging between 0.75 and 2.0 *μ*g/mL. 

 Although all 57 isolates for the 1999-2000 series had been typed, only 36 could be recovered from archives of the 2004 series. From the 100 episodes of bacteraemia in 2006, two MRSA and one MSSA isolate were not saved, allowing 97 isolates to be typed. No significant changes in the lineages of *S. aureus* causing bacteraemia occurred between 1999 and 2006, although the proportion of MRSA bacteraemias caused by CC30 (E-MRSA16) increased progressively from 36.6% to 54%, while the contribution of CC22 (E-MRSA15) decreased from 50% to 38% (Table 3). 

 Combining data from the retrospective and prospective series yielded a total of 195 episodes of bacteraemia for which outcome and antibiotic resistance data were available. Of these, 109 (55.6%) were MRSA bacteraemias, and 49 (25.1%) were complicated cases. Methicillin resistance was not associated with complicated disease (25.7% vs. 24.4%, *P* = .87) but was associated with excess mortality (46.8% vs. 30.2%, *P* = .02). MRSA bacteraemia was associated with a significant excess of diabetic patients (30.1% vs. 8.3%, *P* = .005) and inappropriate initial antibiotic treatment (40.3% vs. 10.4%, *P* = .01). 

 For 152 cases full clinical and typing data were available. These data show no association between methicillin resistance or staphylococcal lineage and complicated bacteraemia (Table 4). 

 Toxin genotyping demonstrated that 3/188 were PVL positive (1.6%) and other gene frequencies were: *tst* 83/188 (44.1%), *sea* 66/188 (35%), and *sej* 4/188 (2.1%). There were no differences in the frequency of these toxin genes between the three time-series. There was no relationship between toxin genotype and complicated disease or mortality at three months. However, all CC30 strains were *tst* positive and all four *sej* positive isolates were of CC5 (Table 5).

## 11. Discussion

The importance of patient and management factors in determining outcome of *S. aureus* bacteraemia is well established [3, 5]. However, while a wide range of bacterial toxin and virulence factors is variably present in *S. aureus *[15] the relationship between strain variation and outcome of *S. aureus* infection is not clear. Peacock et al. reported an association between seven variable toxin and adhesion genes and invasive disease [6], while Lindsay et al. found no association between putative virulence genes and invasive disease using a microarray approach [7]. Fowler et al. studied 324 patients with catheter-related SAB and identified methicillin resistance and the presence of *sea* as risk factors for complicated disease [8]. Lalani et al. evaluated the relationship between bacterial genotype and outcome of 230 episodes of SAB in a phase III clinical trial [16]. In addition to geographical differences in distribution of putative virulence factors, relationships between lineage and clinical presentation were identified. In this study persistence of MRSA bacteraemia after treatment was associated with *seg* and negatively associated with PVL. Ganga et al. found a relationship between SCCmec type and outcome in a study of 253 episodes of SAB, with SCCmec II being an independent predictor of mortality and SCCmec IVa of metastatic infection [17]. 

 The population structure of human* S. aureus* comprises 10 major lineages, or clonal complexes, which have evolved independently [7]. Exchange of genetic material between lineages occurs infrequently, probably because of restriction modification systems [18]. Consequently, if strain differences are important in determining whether bacteraemia is complicated by secondary sites of infection or relapse, then this is likely to be apparent as an association with lineage. However, in our study of 152 cases of SAB we have found no evidence of a relationship between lineage and the development of complicated disease. Nor did we find any association between complicated disease and the presence of PVL, *tst*, *sea*, or *sej* toxin genes. The presence of *tst* gene was strongly related to CC30 (EMRSA-16) which is consistent with previous studies [6, 7, 19]. However in contrast to the findings of Holtfreter et al. who identified 93.3% of *sej* gene positive bacteraemic strains in CC8 complex and 6.7% in CC45, we have found *sej* only in CC5 [19]. Our results uphold their hypothesis that superantigen genes have a strong association to clonal complex, and the most likely explanation for the lineage-specific differences relates to geographical variation. 

 In contrast with Fowler's study [8] we have found no relationship between complicated disease and methicillin resistance. But, in keeping with previous studies recently subjected to meta-analysis, we have found methicillin resistance to be associated with excess mortality [20]. This is likely to be related to higher rates of comorbid disease in these patients, but could also be due to the increased failure rate of MRSA therapy compared to MSSA [20]. 

 Reported figures for the rate at which SAB is complicated by metastatic infection or relapse vary widely between studies. Fowler et al. reported a relapse rate of 12.7% and a metastatic infection rate of 30.3% in North Carolina [2]. In contrast, the UK experience of SAB has consistently been that complicated disease is less common. Among 147 cases from Birmingham, Das et al. reported relapse and metastatic infection rates of 6.5% and 7% [21]. Among 815 cases from Essex, Melzer et al. found a metastatic infection rate of 6.6% [22]. In our study, rates varied over time. Among the patients in the 1999-2000 series for whom we were able to get fully evaluable outcome data, the rate of complicated disease was only 9.1% compared with 27.4% and 27.0% in the 2004 and 2006 series, respectively. This difference is likely related to the small number of diabetics and IDUs in the 1999-2000 series which could reflect genuine differences in the clinical work-load at the trust but should be interpreted with caution since only a small proportion of cases were evaluable. Another potential explanation would have been the emergence of a new virulent clone of *S. aureus* over time but comparison of the lineages from 1999, 2004 and 2006 indicates that no new strain emerged, but that there was a gradual increase of EMRSA-16 (CC30). 

 Previous studies have reported increases in vancomycin MIC to be associated with treatment failure in SAB [23] however we have found no evidence of raised vancomycin MIC. Indeed we have previously reported an apparently paradoxical relationship between the clinical outcome of SAB and the MIC of vancomycin in these patients [24]. 

 Our study has a number of important limitations. It is relatively small and from a single centre and thus does not have the power to detect modest associations between bacterial lineage and outcome. By being observational, patients were not subject to a standardised approach of diagnosis and treatment. For example only 43% of subjects had an echocardiogram and since follow-up blood cultures are rarely performed at our institution in the management of SAB we were unable to assess persistence of bacteraemia on treatment. For these reasons, we may have underestimated the number of complicated cases. However, the role of echocardiography in cases of SAB considered to be at low risk of endocarditis is controversial [25] and having followed-up each case for three months, we feel we are unlikely to have misclassified any complicated cases. In looking at the role of genotype we have combined our robust prospectively gathered data with the less complete retrospectively gathered data set. While being not ideal this has allowed us to more confidently exclude a major role for bacterial genotype in determining outcome and is unlikely to have introduced any significant element of bias. We have classified any second episode of SAB within three months as a relapse rather than considering the possibility of reinfection with a different strain. While this could reduce the power of our study to detect an impact of genotype on outcome, the number of relapses was small so this is also unlikely to be a significant effect. 

 The major strength of our study is that it places the role of bacterial genotype in the context of clinical factors. In contrast with the findings of Fowler and Lalani we have not found a relationship between genotype and outcome [8, 16]. It is likely this reflects differences in patient selection, geographical differences and sample size. Our failure to find evidence of a relationship between bacterial genetic factors and bacteraemia outcome in the 152 cases we have analysed demonstrates that any impact of microbial factors is likely to be small compared to the impact of host and management factors. 

 The associations we have found, duration of symptoms prior to adequate antibiotic therapy, delay in removal of a source of bacteraemia and community onset of symptoms indicate that duration of bacteraemia is the central determinant of risk of complicated disease. Patients who have community onset of symptoms, including IDUs are likely to present later than hospitalised patients. Injecting drug use and diabetes have been identified by some previous studies as risk factors for mortality from SAB [26–28]. The only study we are aware of looking specifically on the impact of diabetes on SAB found an increased rate of endocarditis compared with nondiabetic patients but no difference in mortality [29]. 

 In summary, we have confirmed the previously described risk factors for complicated SAB; community onset of symptoms, diabetes mellitus, delay in initiation of effective antibiotics and failure to identify and remove a primary focus of infection and failed to identify any role for bacterial genotype. Our findings underscore the importance of clinical assessment of patients with SAB for risk of complicated disease and indicate that clinical rather than bacterial factors are the major determinants of outcome in this disease.

## Figures and Tables

**Table 1:** Clinical Characteristics of 100 cases of* S. aureus *bacteraemia identified prospectively from 2006 to 2007.

Clinical Characteristics	Total	Uncomplicated	Complicated	RR	95% CI	*P*
	*n* = 100	*n* = 73	*n* = 27			
Age						
Median	65.5	67	61	—	—	.386*
(with IQR)	(46–78)	(42–77)	(49–78)	—	—	

Male Gender	59 (%)	40 (54.8%)	19 (70.4%)	1.28	0.94–1.50	.096

Patient Location						
Admission Unit	27(%)	**14 (19.2%)**	**13 (48.1%)**	1.56	1.07–2.28	**.006**
ICU	11(%)	10 (13.7%)	1 (3.7%)	0.78	0.62–0.98	.280
Renal Unit	19(%)	16 (21.9%)	3 (11.1%)	0.84	0.66–1.06	.265
Medical Wards	30(%)	25 (34.2%)	5 (18.5%)	0.82	0.65–1.03	.148
Surgical Wards	13(%)	8 (10.9%)	5 (18.5%)	1.21	0.78–1.90	.329

Community Acquired	7(%)	4 (5.5%)	3 (11.1%)	1.30	0.68–2.49	.384

Community Onset	33(%)	**19 (26.0%)**	**14 (51.9%)**	**1.40**	**1.02**–**1.92**	**.018**

Comorbidities						
Diabetes Mellitus	20(%)	**10 (13.7%)**	**10 (37.3%)**	**1.58**	**1.00**–**2.48**	**.021**
IDU	7(%)	**1 (1.4%)**	**6 (22.2%**)	**5.42**	**0.88**–**33.49**	**.001**
Smoker	22(%)	13 (17.8%)	9 (33.3%)	1.30	0.90–1.88	.109
Excess Alcohol	11(%)	9 (12.3%)	2 (7.4%)	0.88	0.65–1.20	.722
HIV	0	0	0	—	—	—
Haemodialysis	17(%)	14 (19.2%)	3 (11.1%)	0.86	0.67–1.12	.55
Central line in situ	43(%)	**38 (52%**)	**5 (18.5%)**	**0.69**	**0.55–0.88**	**.003**
Recent Antibiotics	26(%)	21 (28.8%)	5 (18.5%)	0.87	0.69–1.11	.442
Recent Steroid Therapy	12(%)	9 (12.3%)	3 (11.1%)	0.97	0.68–1.38	1.000
Recent Immunosuppression	6(%)	4 (5.5%)	2 (7.4%)	1.10	0.62–1.96	.660

Management						
Primary Focus Removed						
<72 hours	41(%)	**36 (49.3%)**	**5 (18.5%)**	0.71	0.57–0.90	**.006**
>72 hours/not removed	36(%)	**22 (30.1%)**	**14 (51.8%)**	1.30	0.98–1.74	**.061**
Not identified	23(%)	15 (20.5%)	8 (29.6%)	1.16	0.83–1.60	.423
Initiation of effective antibiotics ≥ 48 hours	32 (%)	**18 (24.7%)**	**14 (51.9%)**	2.10	1.22–3.61	**.015**
Duration of IV treatment >2 weeks	66(%)	47 (64.4%)	19 (70.4%)	1.07	0.84–1.37	.641

Outcome						
Mortality (all cause at 30 days)	38/97**	24/73 (32.9%)	14/25** (56%)	1.29	0.99–1.70	.057
	(39.2%)					

*P-value adjusted for ties. **two patients with complicated bacteraemia were lost to follow up at 30 days.

**Table 2:** Comparison of clinical characteristics of *S. aureus *bacteraemia cases identified retrospectively from 1999-2000 and 2004. Completely evaluable case notes were available for 22 and 73 cases, respectively.

Clinical Characteristics	1999-2000 series	2004 series	*P*
	*n* = 57	*n* = 116	
Age			
Median (interquartile range)*	63.1 (53–78)	68.85 (52–80)	.23

Male Gender	38 (66.7%)	81 (69.8%)	.4

Patient location			
Admission unit	8/47 (17.0%)	18/112 (16.1%)	.53
ICU	8/47 (17.0%)	19/112 (16.9%)	.58
Renal unit	8/47 (17.0%)	26/112 (23.2%)	.26
Medical wards	17/47 (36.2%)	32/112 (28.6%)	.22
Surgical wards	6/47 (12.8%)	17/112 (15.2%)	.45

Community acquired	2/22 (9.1%)	4/73 (5.5%)	.42

Community onset	5/22 (22.7%)	21/73 (28.7%)	.39

Diabetes Mellitus	2/22 (9%)	20/73 (27.4%)	.06

IDU	0/22 (0%)	7/73 (9.6%)	.15

Smoker	6/22 (27.3%)	19/73 (26.0%)	.55

Central line *in situ *	10/22 (45.5%)	39/73 (53.4%)	.3

**Table 3:** Lineages of 190* S. aureus* bacteraemia isolates from 1999-2000, 2004, and 2006.

Clonal Complex	MSSA						MRSA					
	1999-20000		2004		2006-2007		1999-2000		2004		2006-2007	
	N	%	N	%	N	%	N	%	N	%	N	%
CC22	2	7.4	2	13.3	4	8.5	15	50.0	10	47.6	19	38
CC30	7	25.9	3	20.0	10	21.3	11	36.6	8	38.1	27	54
CC45	6	22.2	3	20.0	11	23.4	0	0	0	0	0	0
CC1	1	3.7	1	6.7	6	12.8	0	0	0	0	4	8
CC8	1	3.7	0	0	5	10.6	2	6.7	0	0	0	0
CC5	3	11.1	0	0	4	8.5	2	6.7	0	0	0	0
Other	7*	25.9	6^†^	40.0	7^¶^	14.9	0	0	3**	14.3	0	0
Total	27	100	15	100	47	100	30	100	21	100	50	100

*comprising MLSTs 6, 12(x2) 15, 123(x2), and 188, ^†^comprising MLSTs 12, 15, 20, 97, 101, and 123, ^¶^not MLSTed. **comprising MLST12 (x3). CC=clonal complex.

**Table 4:** Lineages of 152 *S. aureus *isolates from episodes of uncomplicated and complicated bacteraemia.

Clonal complex	Total	Uncomplicated	Complicated	RR	95% CI	*P*
	*n* = 152	*n* = 116 (76.3%)	*n* = 36 (23.7%)			
CC30	53	42 (79.2%)	11 (20.8%)	0.94	0.79–1.13	0.689
CC22	39	29 (74.4%)	10 (25.6%)	1.04	0.84–1.28	0.828
CC45	15	11 (73.3%)	4 (26.7%)	1.05	0.76–1.44	0.755
CC1	11	7 (63.6)	4 (36.4%)	1.22	0.77–1.92	0.291
CC8	7	6 (85.7%)	1 (14.3%)	0.88	0.65–1.21	1.00
CC5	7	6 (85.7%)	1 (14.3%)	0.88	0.65–1.21	1.00
Other	20	15 (75%)	5 (25%)	1.02	0.78–1.34	1.00

**Table 5:** The distribution of toxin genes within the 6 dominant *S. aureus *lineages.

Clonal Complex	Toxin Gene			
	PVL *n* = 3	*tst-1 * *n* = 83	*sej * *n* = 4	*sea * *n* = 66
CC30	0	71.1%	0	63.6%
CC22	33.3%	9.6%	0	1.5%
CC45	0	2.4%	0	1.5%
CC1	0	1.2%	0	9.1%
CC8	0	1.2%	0	9.1%
CC5	0	3.6%	100%	6.1%
Other	66.6%	10.8%	0	9.1%
